# Supplementary material for: Phototropin connects blue light perception to starch metabolism in green algae
Source: Nat Commun. 2025 Mar 15;16:2545. doi: 10.1038/s41467-025-57809-3 (PMC11909140; doi:10.1038/s41467-025-57809-3)
Supplement: Supplementary file 1 — Supplementary information [file 41467_2025_57809_MOESM1_ESM.pdf]

Supplementary Materials for

Phototropin connects blue light perception to starch metabolism in green algae

Yizhong Yuan[1,†], Anthony A Iannetta[2], Minjae Kim[3], Patric W. Sadecki[2], Marius Arend[4,5,6], Angeliki Tsichla[1, ††], M. Águila Ruiz-Sola[1,†††], Georgios Kepesidis[1, ††††], Denis Falconet[1], Emmanuel Thevenon[1], Marianne Tardif[7], Sabine Brugière[7], Yohann Couté[7], Jean Philippe Kleman[8], Irina Sizova[9], Marion Schilling[1], Juliette Jouhet[1], Peter Hegemann[9], Yonghua Li-Beisson[3], Zoran Nikoloski[4,5,6], Olivier Bastien[1], Leslie M. Hicks[2], Dimitris Petroutsos[1,10]*

Corresponding author: Dimitris Petroutsos, dimitris.petroutsos@ebc.uu.se

This file includes:

Supplementary Text
Supplementary Figs. 1 to 24
Supplementary References

Supplementary Text

Phylogenetic analysis

PMSK1 is found to belong to a family of Serine/threonine-protein kinases (named the PMSK-like family, see material and methods) which is conserved in green algae and vascular plants (**Supplementary Figs. 21 and 22**). In particular, the PMSK-like family is consistent with the known Chlorophyta evolution [1]. Interestingly, when searching for PMSK1 similar sequences into the *Arabidopsis thaliana* genome in the NCBI database [2], next to the PMSK-like *Arabidopsis* members come the *Arabidopsis* HT1 (AT1G62400, NP_176430.2), CBC1 (AT3G01490, NP_186798.1) and CBC2 (AT5G50000, NP_199811.1). It has been found that (i) the best reciprocal best hit for these three proteins when searching for *Chlamydomonas reinhardtii* was PMSK-like family members, especially PMSK1 (ii) this was true when searching for any Chlorophyta species and that (iii) regarding into the three family CBC, HT and PMSK-like, Chlorophyta was only present in the latest (**Supplementary Fig. 23**). A simple molecular clock analysis phylogenetic analysis revealed that the root is likely to be placed between the CBC and the HT/PMSK1 family (**Supplementary Fig. 24**) suggesting that a representative for these three families could have been present in the common ancestor of the Chlorophyta. One explanation for the observed distribution of the sequences is that both HT and CBC representative have been lost before the radiation of the Chlorophyta. It must be noticed that most of the time, duplication in CBC, HT and PMSK-like family seems to have happened after recent speciation events, suggesting that a few members of this family could have been present in the last common ancestor of the Chlorophyta/Charophyta/Land Plants Phylum.

Identification of the PMSK-like family

The gene product of Cre16.g659400, identified as encoding a Ser/Thr protein kinase (**Fig. 3b**) and named kinase Phototropin-mediated signalling kinase 1 (PMSK1), was translated and the amino acid sequence used as a query for a series of BLAST searches in the publicly available National Center for Biotechnology Information (NCBI) database [2]. Both BLASTp and PSI-BLAST [3,4] were performed together with a human-curated process in order to obtain the widest dataset possible and the most robust one. The final dataset (#1) contained 111 amino acid sequences from at least 10 orders of Chlorophyta, Charophyta, Bryopsida, Pteridophyta, Gymnospermae and Angiosperm.

Identification of a relation between the PMSK-like family and both the HT1 and the CBC family

Because both HT1, CBC1 and CBC2 were identified in the BLAST research as first remotely related *Arabidopsis thaliana* sequences in the previous homologous sequences search, a reciprocal best hit research was done using these three sequences together with a human-curated process and results was append to the previous dataset. This new dataset (#2) contained 278 amino acid sequences from at least 10 orders of Chlorophyta, Charophyta, Bryopsida, Pteridophyta, Gymnospermae and Angiosperm. Interestingly, homologous sequences investigations revealed that while the four family Charophyta, Bryopsida, Pteridophyta, Gymnospermae are present in the both the HT and the CDC family, the most closely related Chlorophyta sequences of the CBC/HT *Arabidopsis* thaliana representative were actually the PMSK-like family sequences. A subset of dataset #2 containing 11 sequences was used to compute both a clock-model and a relaxed-clock model to infer the possible root of the three PMSK-like, HT and CBC family.

Multiple Sequence Alignments

Amino acid multiple sequence alignments for each protein segment were created using MUSCLE [5] and then adjusted using Gblocks (version 0.91.1) [6] or the MEGA ML analysis (see below) or manually using Jalview (version 2.11.1.4) [7] for both the PhyML and the Bayesian analysis (see below).

Dataset #1 and #3. Bayesian Inferences

Two independent Metropolis-coupled Markov Chain Monte Carlo (MCMC) analyses were performed using the final curated datasets in the MrBayes (version 3.2.7.a) software [8]. The two datasets were used for Bayesian inference. The first was include in a nexus file resulting from the MUSCLE alignment. The dataset #3 was include in the 278 sequences multiple alignment nexus file from the dataset #2 from which, following a command line in the MrBayes software, it was extracted before setting the algorithm parameters. The Bayesian posterior probabilities (BPP) were estimated by two independent runs of four Metropolis Coupled chains (MCMC): 2 independent runs, together with one cold chain and three "heated" (temperature parameter 0.1, Dataset 3) chains drove the analyses. The analyses were allowed to switch among all the substitution models implemented in the software to identify the best model without any a priori (aamodelpr = mixed). The prior on the branch length was set to unconstrained for the dataset #1, leading to an unrooted tree whereas it was set on both a uniform and a relaxed clock model to infer the root position of the HT, CBC and PMSK-like families.
80,000 and 160,000 generations with sampling every 100 generations were set for Dataset #1 and Dataset #3, respectively. The standard deviation of the split frequency between the two parallel analyses at the end of each analysis were 0.021973 and 0.005348 for Dataset #1 and Dataset #3, respectively. The first 25% of the trees produced were discarded in order to let the analyses stabilize (burnin = 0.25). The convergence of the runs was estimated and the potential scale reduction factor (PSRF +) were all around 1 for all the parameters indicating that the associated chains converged to one target distribution. Large PSRF (PSRF + >1) indicate convergence failure. Nodes with a posterior probability ≥0.90 were considered as well supported. Alignments in fasta and trees in newick format are available upon request.

Dataset #2. Maximum Likelihood inferences

Unrooted maximum likelihood (ML) phylogenetic trees were then inferred using both the MEGA (version 11) software package Whelan And Goldman ML model [9] and PhyML (version 3.3.1) [10] based on the best-fit models of amino acid substitution determined by Find Best Protein Model [7]. Non-uniformity of evolutionary rates among sites was modeled using a discrete Gamma distribution (+ G) and a certain fraction of sites were considered to be evolutionarily invariable (+ I). Initial trees for the heuristic search were obtained automatically by applying Neighbor Joining (NJ) and BioNJ algorithms to a matrix of pairwise distances estimated using a JTT model, and then selecting the topology with superior log likelihood value. All positions with less than 95% site coverage were eliminated. Statistical tests for branch support for the MEGA ML algorithm was estimated with a Bootstrap procedure [11], using 100 replicates. Statistical tests for branch support concerning the PhyML algorithm was estimated with the aLRT SH-like branch support method [12]. Trees were drawn to scale with the branch length measured in the number of substitutions per site.

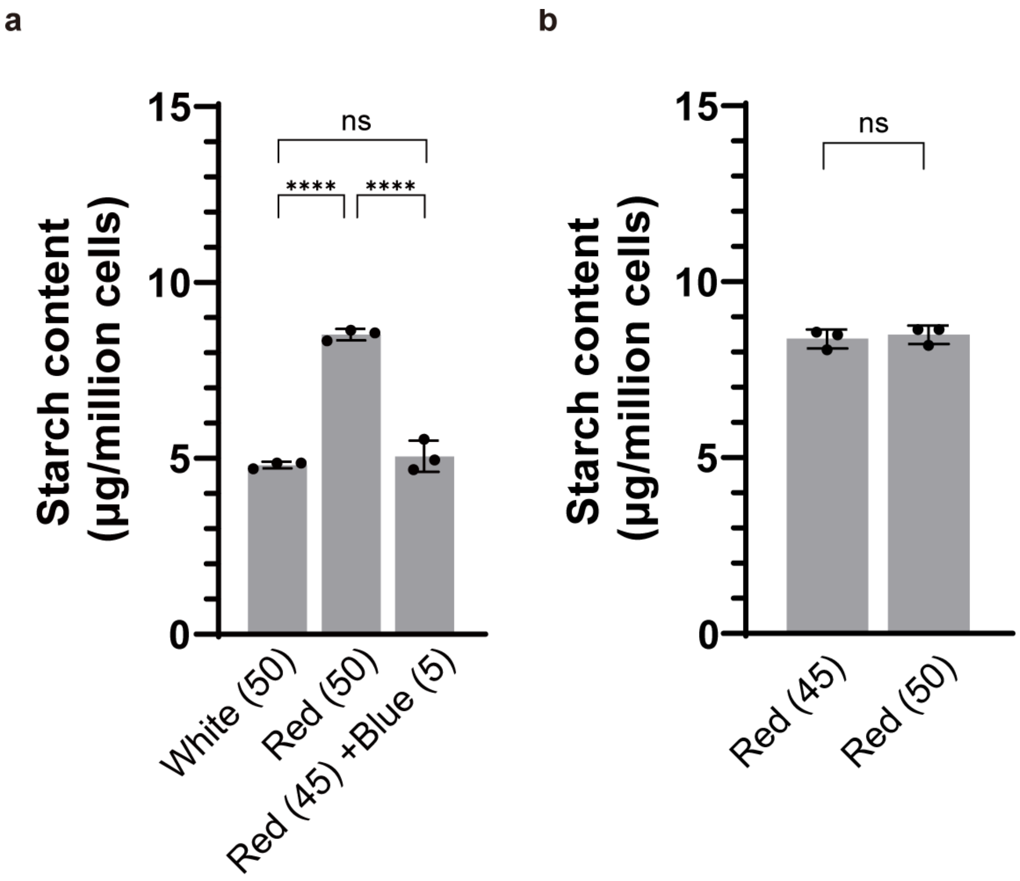

Supplementary Fig. 1. Effect of superimposing low intensity blue light on red light on starch content. a Starch content of WT was measured under continuous light conditions: white light, red light, or red light supplemented with low-intensity blue light. **b** Starch content of WT was also compared under the two different red-light intensities used in (a). Light intensities in µmol photons m^{-2} s^{-1} are indicated in the x-axis. The statistical significance was determined using one-way ANOVA with Dunnett's multiple comparisons tests. Data are represented as mean ± SD (n = 3 biologically independent samples). Asterisks indicated the p-values (****, $p < 0.0001$; ns, not significant)

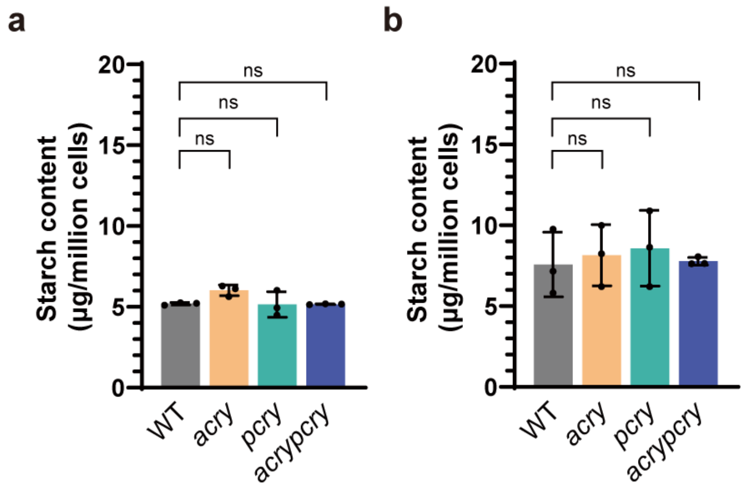

Supplementary Fig. 2. Mutants devoid of animal- or plant-like cryptochromes accumulate WT-levels starch. The starch content of *acry, pcry and acrypcry* synchronized to a 12 h/12 h light dark cycle (white light). Samples were collected at the start (**a**) and at the end of the light phase (**b**). The statistical significance was determined using one-way ANOVA with Dunnett's multiple comparisons tests. Data are represented as mean ± SD (n = 3 biologically independent samples). ns, not significant.

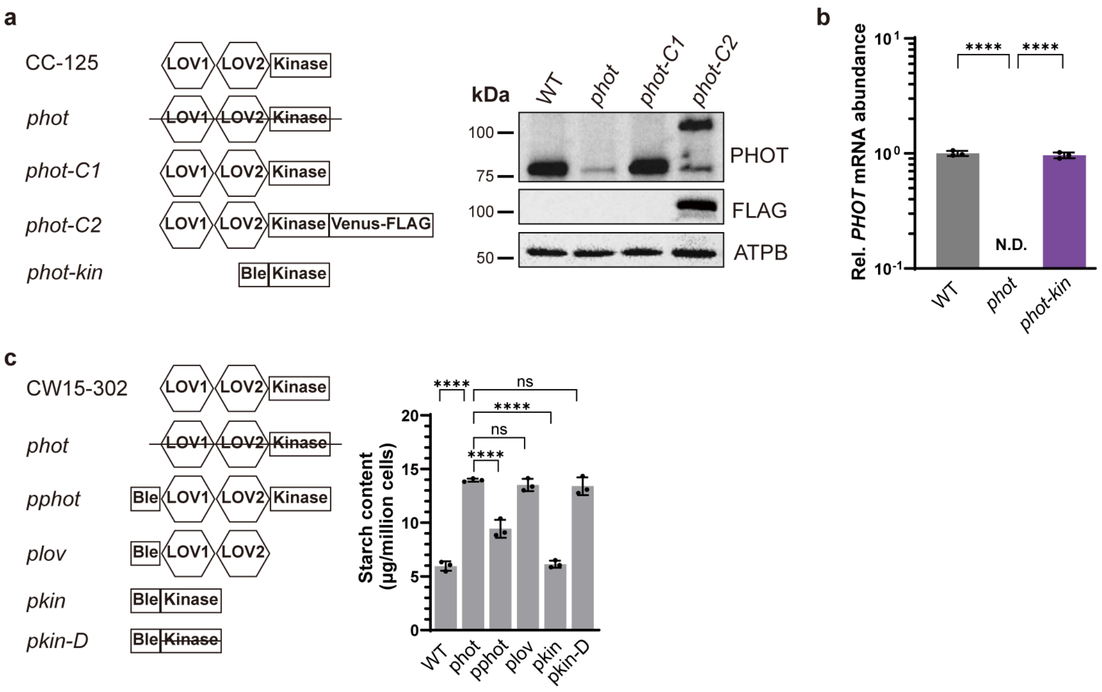

Supplementary Fig. 3. PHOT inhibits starch accumulation in *Chlamydomonas reinhardtii*. a Left: Domains of the *PHOT* gene in WT and complemented *phot* lines. LOV1 and LOV2, photosensory domains; Kinase, kinase domain; Venus-FLAG, yellow fluorescent protein with FLAG tag; Ble, gene conferring resistance to bleomycin. Right: Immunoblot analyses of PHOT level in WT (CC125), *phot* and complements strains. **b**, RT-qPCR analysis of *PHOT-Kinase* transcription level in WT and *phot-kin*. N.D., not detected. **c**, Left: Domains of the *PHOT* gene in WT (*cw15-302*) and complemented *phot* lines. Right: Starch content of WT, *phot* and various *phot*-complemented lines grown under continuous white light. Data are represented as mean ± SD (n = 3 biologically independent samples). The statistical significance was determined using one-way ANOVA with Dunnett's multiple comparisons tests (**b**) of log10 transformed mRNA data as indicated in the graphs. The statistical significance was determined using one-way ANOVA with Dunnett's multiple comparisons tests (**c**). Asterisks indicated the p-values (*, p<0.05; **, p < 0.01; ***, p < 0.001; ****, p < 0.0001; ns, not significant).

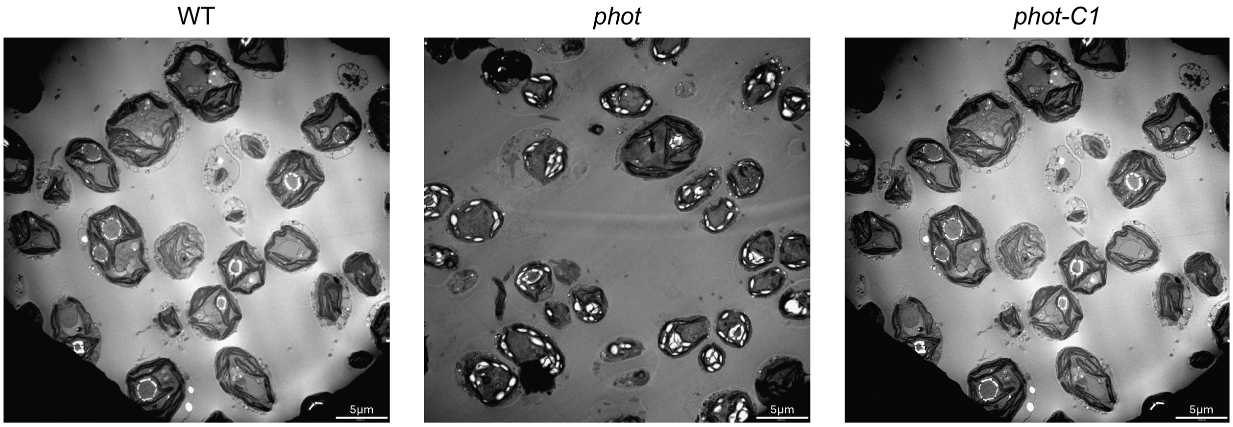

Supplementary Fig. 4. Transmission electron microscopy pictures of WT, *phot,* and *phot-C1*. All strains were synchronized to a 12/12 light dark cycle under white light and samples were collected at the end of the light phase. Scale bars are indicated in the lower-right corner of each panel (5 µm).

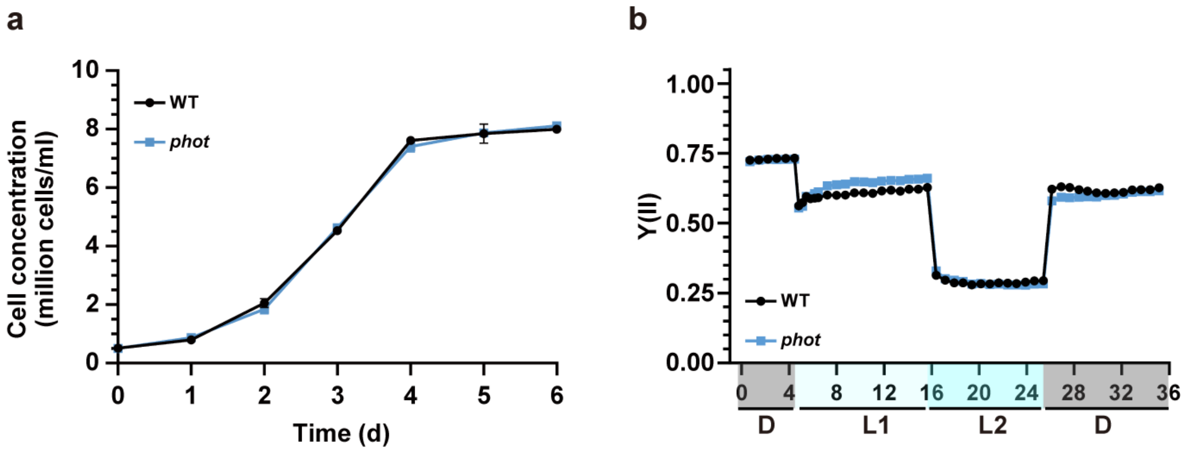

Supplementary Fig. 5. The *phot* mutant shows WT-like growth and photosynthetic efficiency. Growth curves (**a**) and photosystem II efficiency (YII) (**b**) of WT and *phot* grown under continuous white light. *In vivo* chlorophyll fluorescence was recorded in the dark (labelled as "D"), at 21 (labelled as "L1") and 336 (labelled as "L2") µmol photons m^{-2} s^{-1} as indicated in the graphs. Y(II) values calculated as (Fm'-F)/Fm' (n = 3 biological samples, mean ± SD). Please note that in some cases the error bars are smaller than the data point symbols.

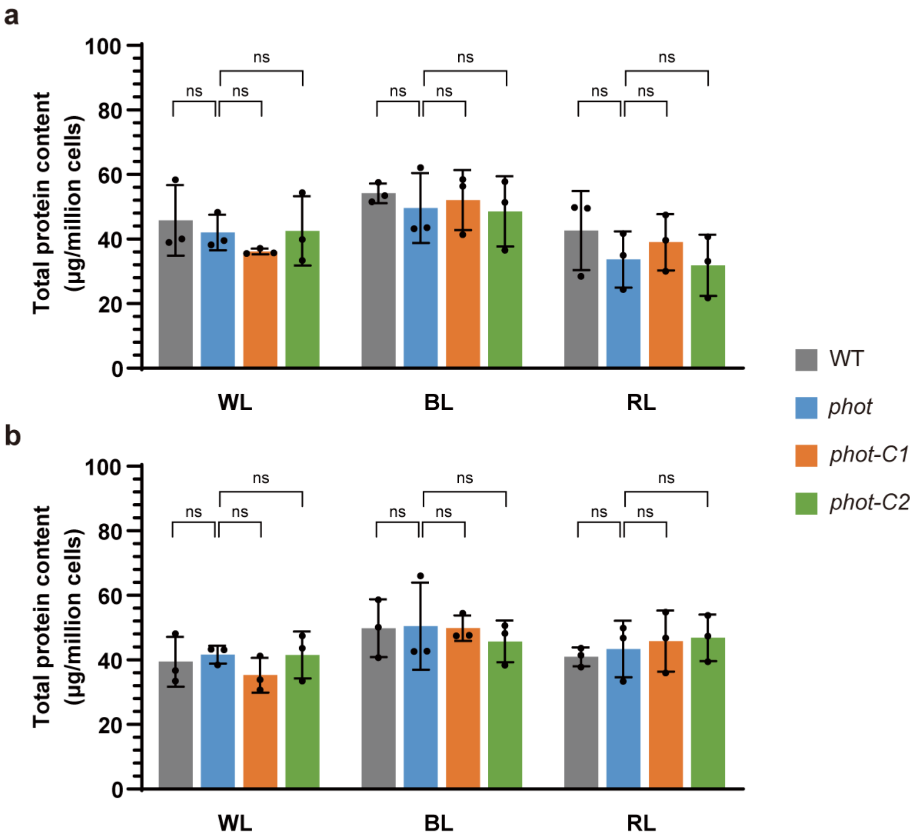

Supplementary Fig. 6. The *phot* mutant has WT-like total protein content. The total protein content of WT, *phot* and complemented *phot* lines synchronized to a 12 h/12 h light dark cycle under different light qualities (WL, white light; BL, blue light; RL, red light). Samples were collected at the start of the start (**a**) and at the end (**b**) of the light phase. The statistical significance was determined using two-way ANOVA with Dunnett's multiple comparisons tests. Data are represented as mean ± SD (n = 3 biologically independent samples). ns, not significant.

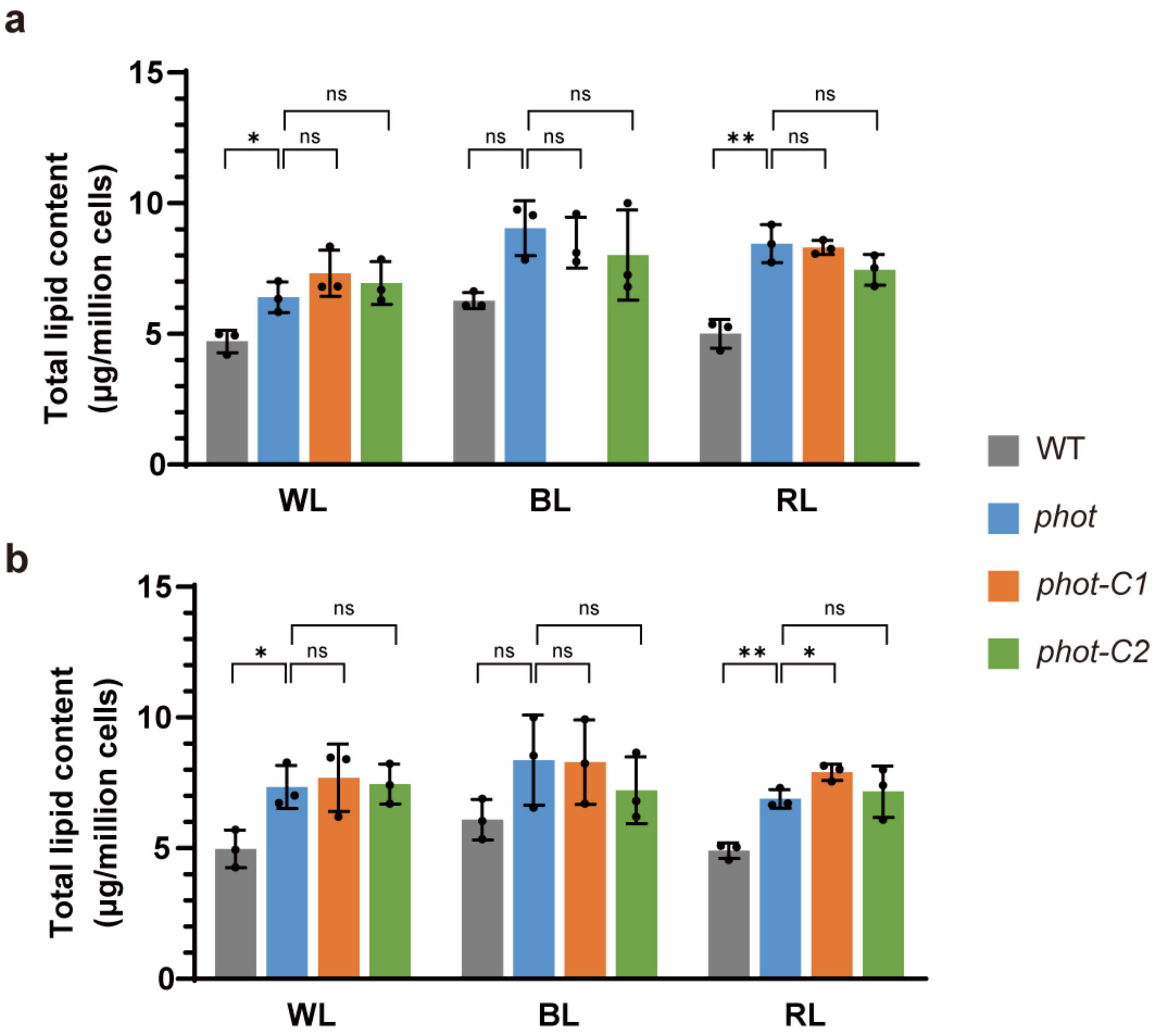

Supplementary Fig. 7. The *phot* mutant has WT-like total lipid content. The total lipid content of WT, *phot* and complemented *phot* lines synchronized to a 12 h/12 h light dark cycle under different light qualities (WL, white light; BL, blue light; RL, red light). Samples were collected at the start of the start (**a**) and at the end (**b**) of the light phase. The statistical significance was determined using two-way ANOVA with Dunnett's multiple comparisons tests. Data are represented as mean ± SD (n = 3 biologically independent samples). Asterisks indicated the p-values (**, p < 0.01; ns, not significant).

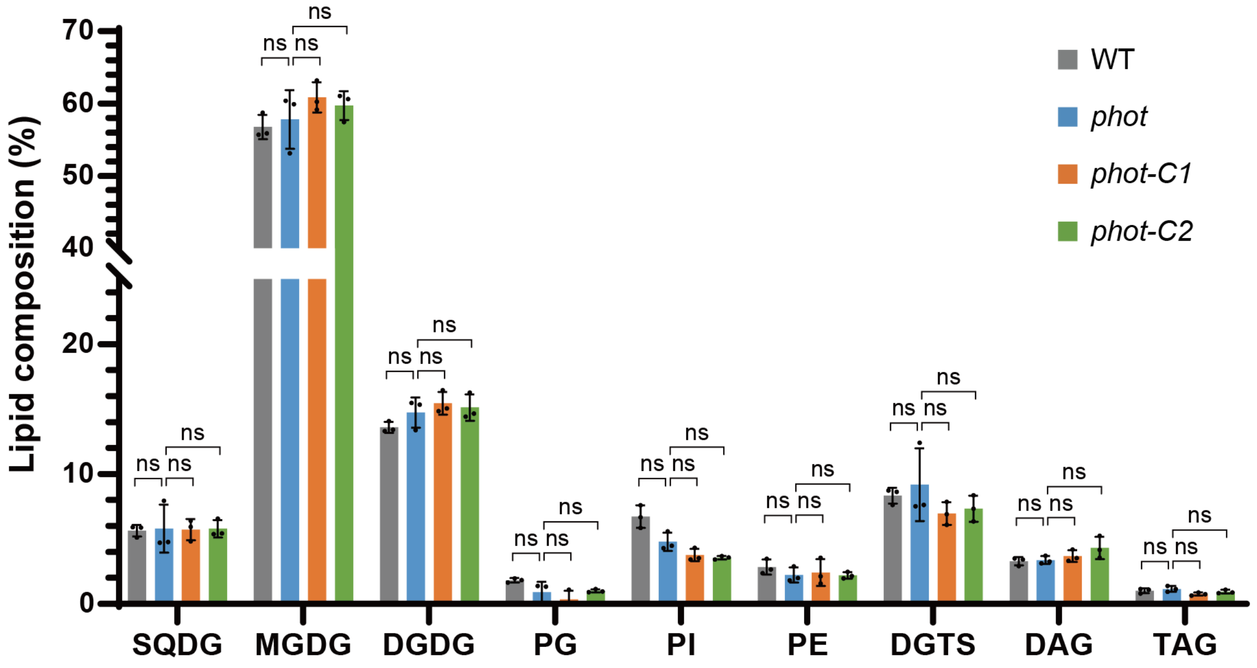

Supplementary Fig. 8. The *phot* mutant has WT-like lipid composition. The lipid composition of WT*, phot and* complemented *phot lines* under 12h/12h light dark conditions. Samples are collected at the end of the light phase. Data are represented as mean ± SD (n = 3 biologically independent samples). SQDG, sulfoquinovosyl diacylglycerol; MGDG, monogalactosyl diacylglycerol; DGDG, digalactosyl diacylglycerol; PG, Phosphatidylgylcerols; PI, Phosphatidylinositols; PE, Phosphatidylethanolamines; DGTS, diacylglycerol-trimethyl homoserine; DAG, diacylglycerols; TAG, triacylglycerols. The statistical significance was determined using two-way ANOVA with Dunnett's multiple comparisons tests. In some cases, the error bars are smaller than the data point symbols. ns, not significant.

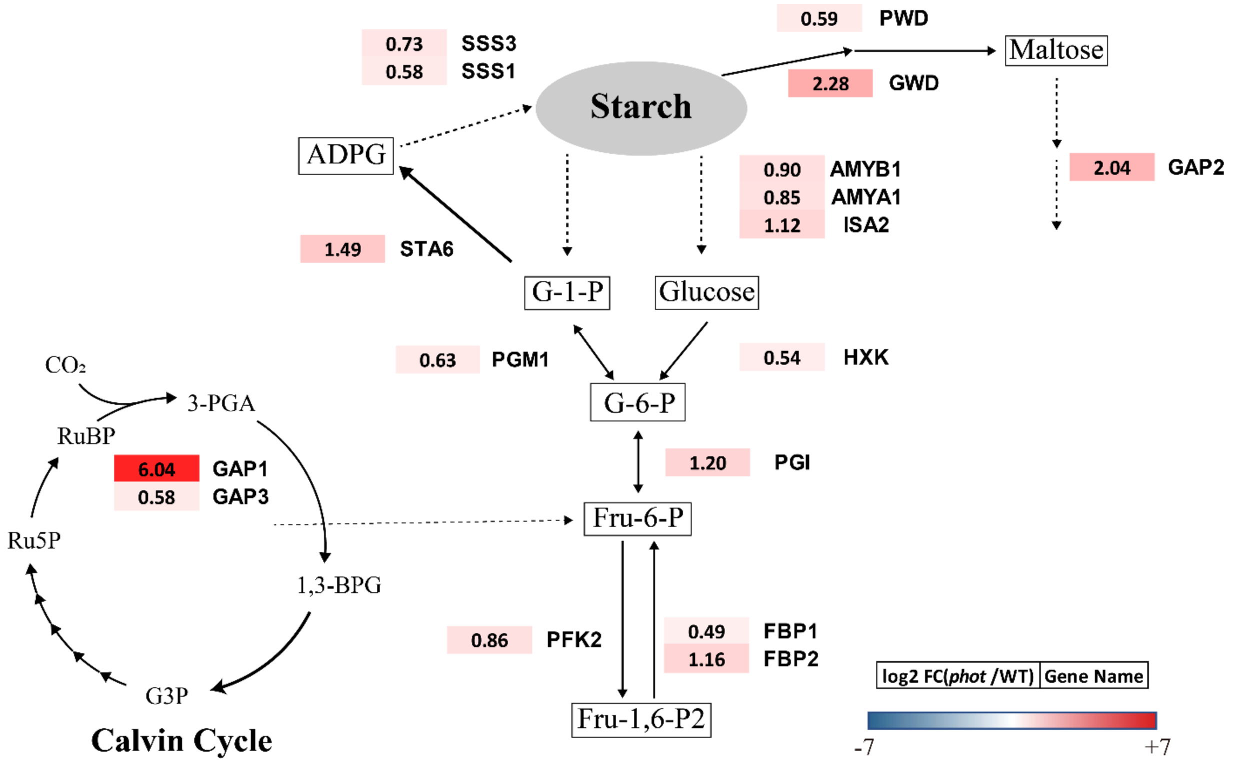

Supplementary Fig. 9. The mRNA abundance of genes involved in starch metabolism is increased in the *phot* mutant. WT and *phot* were synchronized to a 12h/12h light dark cycle under white light. Samples were taken in the middle of the light phase (6h) and the extracted RNA was analyzed by RT-qPCR to quantify mRNA levels of the following genes: GAP, glyceraldehyde-3-phosphate dehydrogenase; PFK, phosphofructokinase; FBP, Fructose-1,6-bisphosphatase; PGI, phosphoglucose isomerase; PGM, phosphoglucomutase; HXK, Hexokinase; STA6, ADP-glucose pyrophosphorylase small subunit; SSS, starch synthase; AMY, α-amylase; ISA, isoamylase; PWD, phosphoglucan water dikinase; GWD, α-glucan water dikinase. Fold change between *phot* and WT was calculated as log2 FC (*phot*/WT).

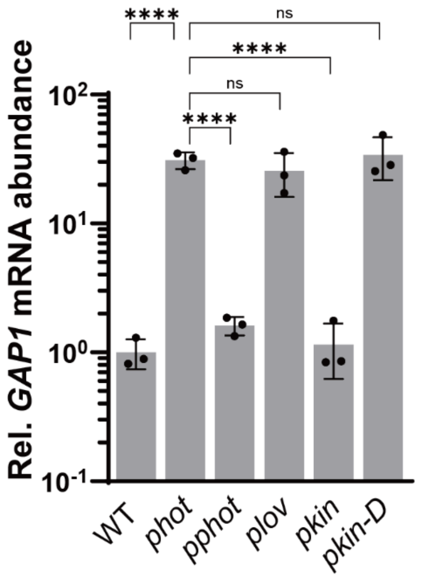

Supplementary Fig. 10. PHOT suppresses *GAP1* mRNA accumulation. *GAP1* relative mRNA abundance in WT (*cw15-302*) and in different *phot*-complemented lines, grown under continuous white light. Data are represented as mean ± SD (n = 3 biologically independent samples). The statistical significance was determined using one-way ANOVA with Dunnett's multiple comparisons tests of log10 transformed mRNA data as indicated in the graphs. Asterisks indicated the p-values (*, p<0.05; **, p < 0.01; ***, p < 0.001; ****, p < 0.0001; ns, not significant).

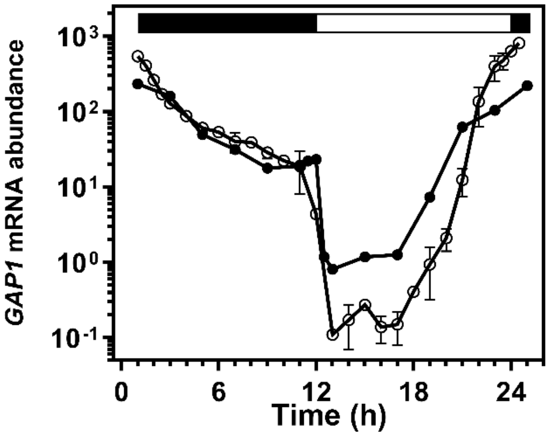

Supplementary Fig. 11. ***GAP1* transcription level reaches its peak at dusk.** *GAP1* expression profile in cells synchronized to a 12h/12h light dark cycle under white illumination, from *Strenkert et al.* (ref. 28, closed circles) and *Zones et al.* (ref. 27, open circles). The dark phase is indicated by black bars above the graphs; the light phase by white.

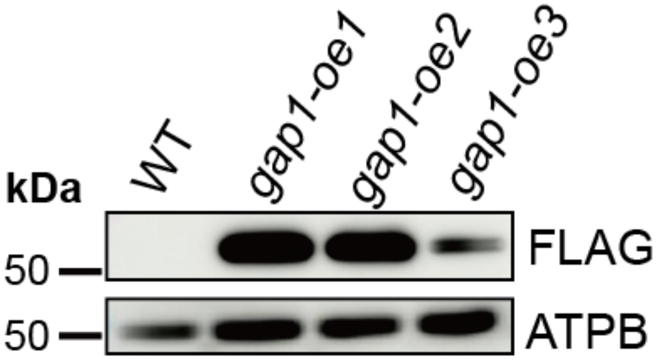

Supplementary Fig. 12. **Immunoblot analyses of *gap1-oe* lines.** Samples were collected from cultures grown in mixotrophic medium containing acetate (TAP medium) under continuous white light. Total protein extracts were probed with anti-FLAG antibody to quantify GAP1-FLAG levels. ATPB served as a loading control.

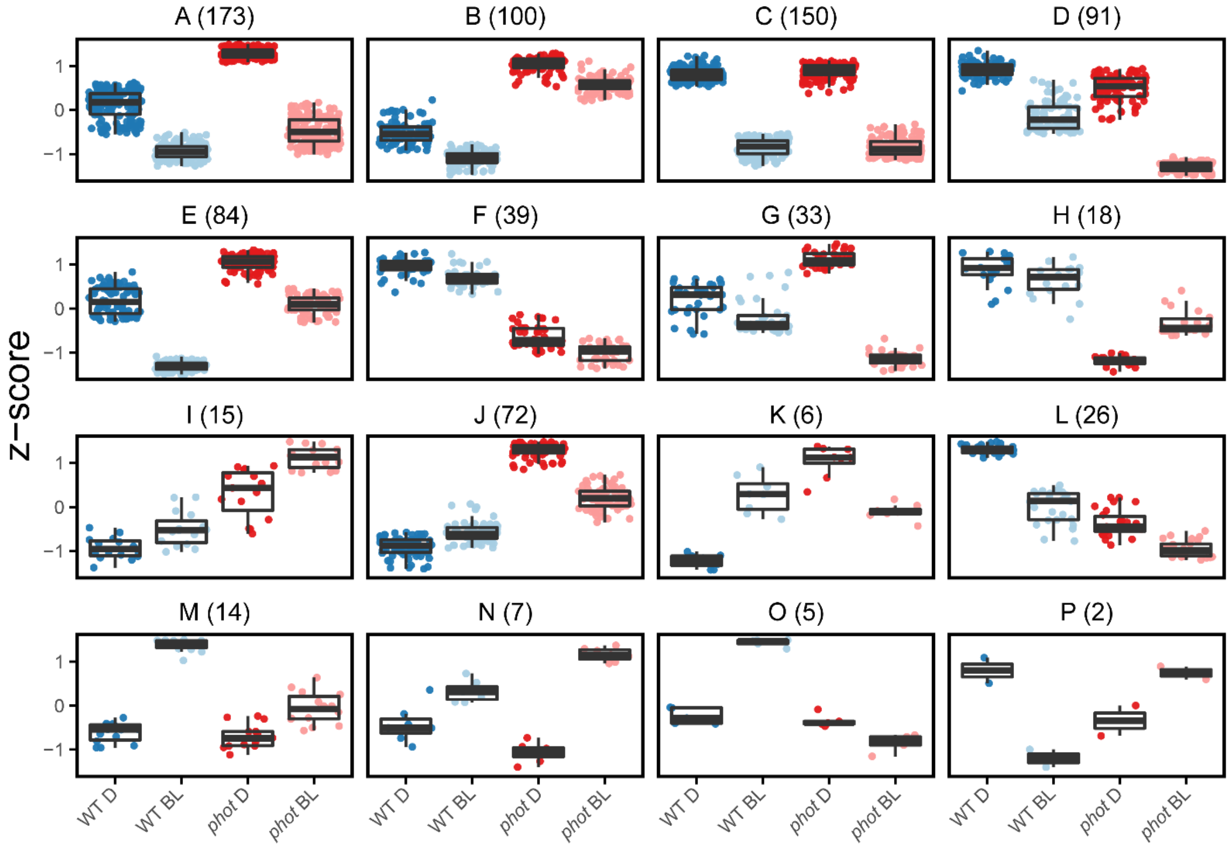

Supplementary Fig. 13. Unsupervised hierarchical clustering analyses of phosphoproteome. Significantly changing phosphopeptides across treatments based on a one-way ANOVA (FDR-adjusted *p*-value < 0.05). Each cluster is labelled A-P, and the number of phosphopeptides contained in each cluster are in parenthesis. Z-scores represent the relative abundance of each peptide across condition.

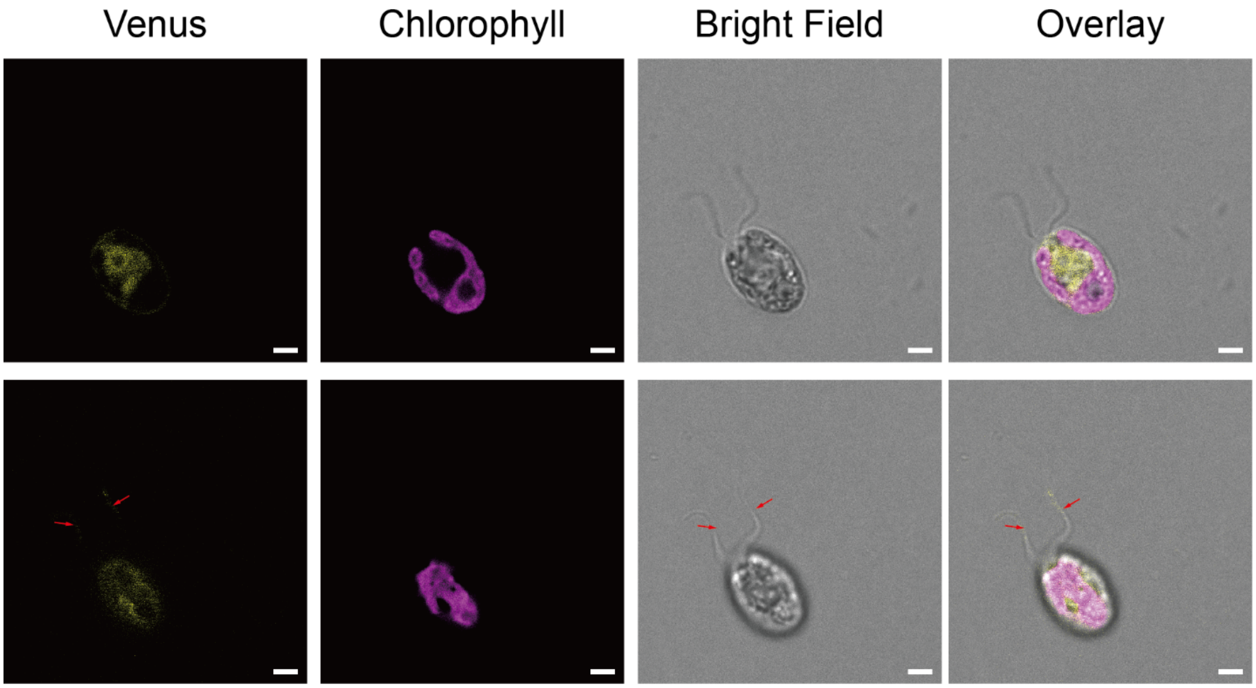

Supplementary Fig. 14. PMSK1 subcellular localization. Representative confocal fluorescent microscopy images PMSK1-Venus (Yellow) constitutively expressed in WT. Scale bar: 2 µm, red arrows indicate flagella. Two focal planes representative of the volume at 2.1 µm in distance were shown.

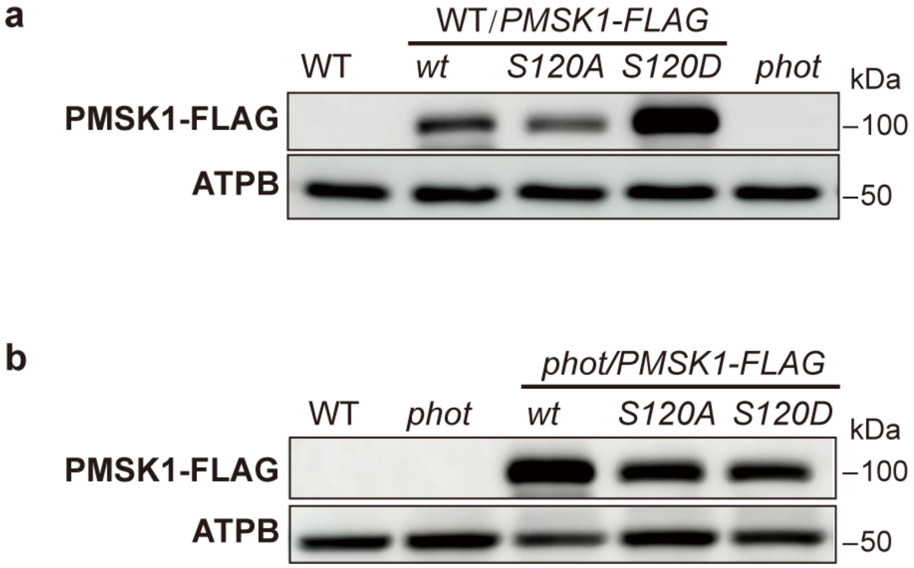

Supplementary Fig. 15. PMSK1-FLAG protein levels in WT/*PMSK1-FLAG* and *phot*/*PMSK1-FLAG* lines. Samples were collected from cultures grown in mixotrophic medium containing acetate (TAP medium) under continuous white light. Total protein extracts were probed with anti-FLAG antibody to quantify PMSK1-FLAG levels in the WT **(a)** or *phot* **(b)** background. ATPB served as a loading control.

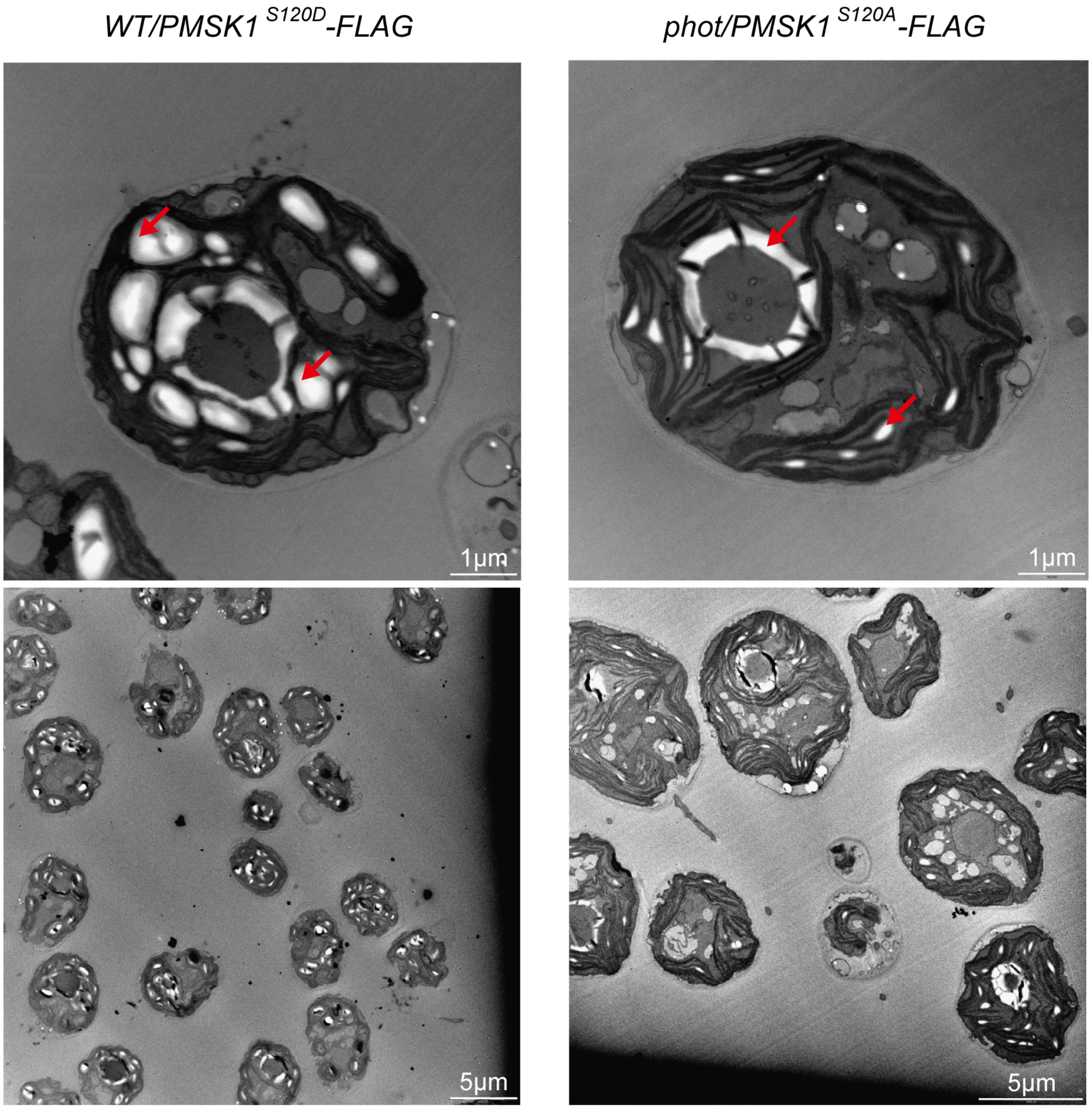

Supplementary Fig. 16. Transmission electron microscopy pictures of WT/*pmsk1*^{S120D} and *phot/pmsk1*^{S120A}. The two strains were synchronized to a 12/12 light dark cycle under white light and samples were collected at the end of the light phase. Top panels: Single cells. Bottom panels: Groups of cells. Scale bars are indicated in the lower-right corner of each panel (1 µm or 5 µm). Red arrows point to starch granules.

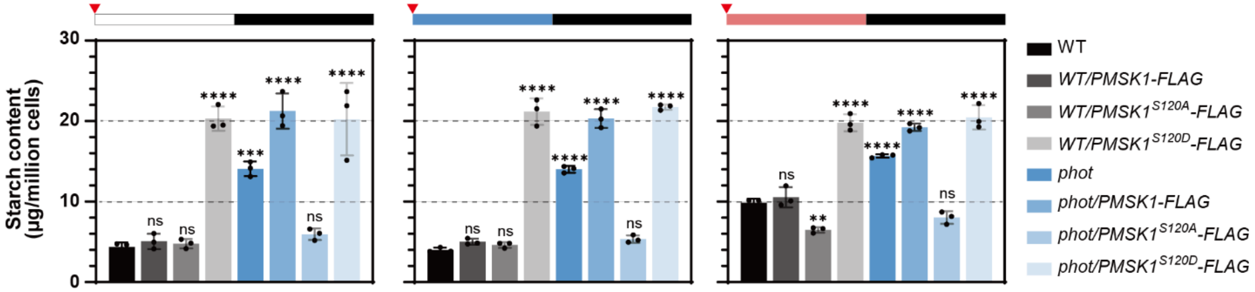

Supplementary Fig. 17. Impact of PMSK1-FLAG overexpression in WT and *phot* on starch metabolism. Starch content in WT or *phot* overexpressing PMSK1-FLAG. Cells were synchronized to a 12h/12h light dark cycle under different light qualities. The dark phase is indicated by black bars above the graphs; the light phase by white, blue or red bars, depending on the light quality used. Red triangles indicate sample collection time. Data are represented as mean ± SD (n = 3 biologically independent samples). The statistical significance was determined using one-way ANOVA with Dunnett's multiple comparisons tests. Asterisks indicate the *P* values compared to WT. (*, p<0.05; **, p < 0.01; ***, p < 0.001; ****, p < 0.0001; ns, not significant). Please note that in some cases the error bars are smaller than the data point symbols.

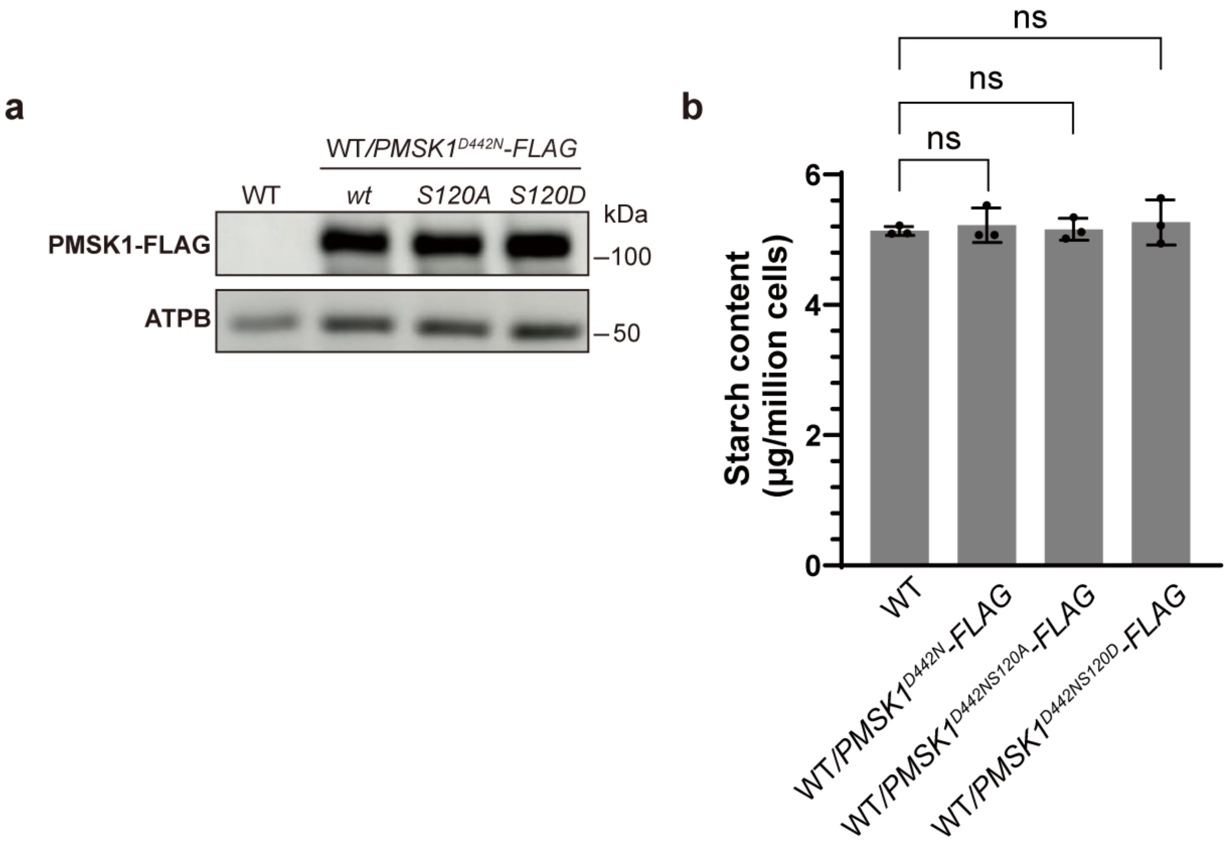

Supplementary Fig. 18. PMSK1 kinase activity is necessary to mediate light quality dependent starch metabolism in *Chlamydomonas reinhardtii* (**a**) Immunoblot analyses of PMSK1-FLAG in various PMSK1-FLAG overexpressing lines. ATPB served as a loading control. (**b**) Starch content of various dead kinase PMSK1-FLAG overexpression lines grown under continuous white light. The statistical significance was determined using one-way ANOVA with Dunnett's multiple comparisons tests. Data are represented as mean ± SD (n = 3 biologically independent samples). ns, not significant.

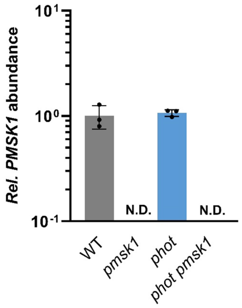

	sgRNA target site	Indel
**WT**	CCGCCCGTTGCAGCTCA---------CCCGGG	-
pmsk1	CCGCCCGTTGCAGCTCA (Paro^R) CCCGGG	+2,089
phot pmsk1	CCGCCCGTTGCAGCTCA (Ins) CCCGGG	+126

Supplementary Fig. 19. *pmsk1* and *phot pmsk1 knockout mutant generation.* Left: PMSK1 relative mRNA abundance in different lines, grown under continuous white light. Data are represented as mean ± SD (n = 3 biologically independent samples). N.D., not detected. Right: Sequence alignment of target site amplicons from mutant strains with the corresponding wild-type sequence. NGG PAM sequences are red highlighted.

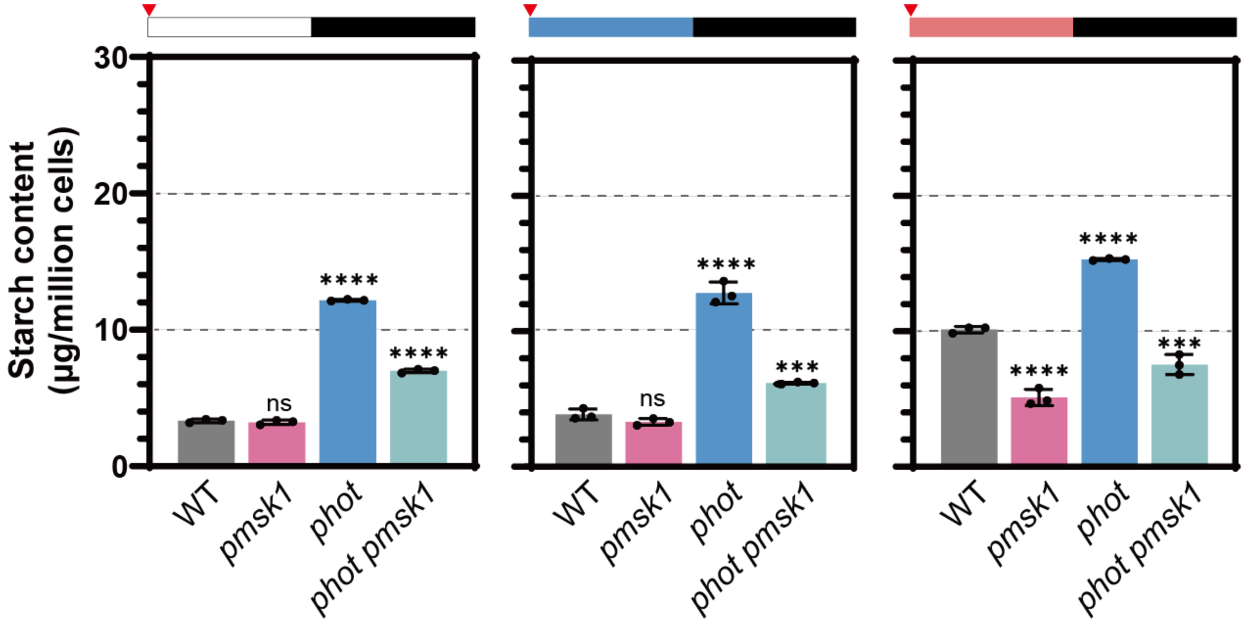

Supplementary Fig. 20. Starch content in WT and in single and double *phot pmsk1* mutants sampled at dawn. Cells were synchronized to a 12h/12h light dark cycle under different light qualities. The dark phase is indicated by black bars above the graphs; the light phase by white, blue or red bars, depending on the light quality used. Red triangles indicate sample collection time. The statistical significance was determined using one-way ANOVA with Dunnett's multiple comparisons tests. Asterisks indicated the p-values compared to WT. (*, p<0.05; **, p < 0.01; ***, p < 0.001; ****, p < 0.0001; ns, not significant). Data are represented as mean ± SD (n = 3 biologically independent samples). Please note that in some cases the error bars are smaller than the data point symbols.

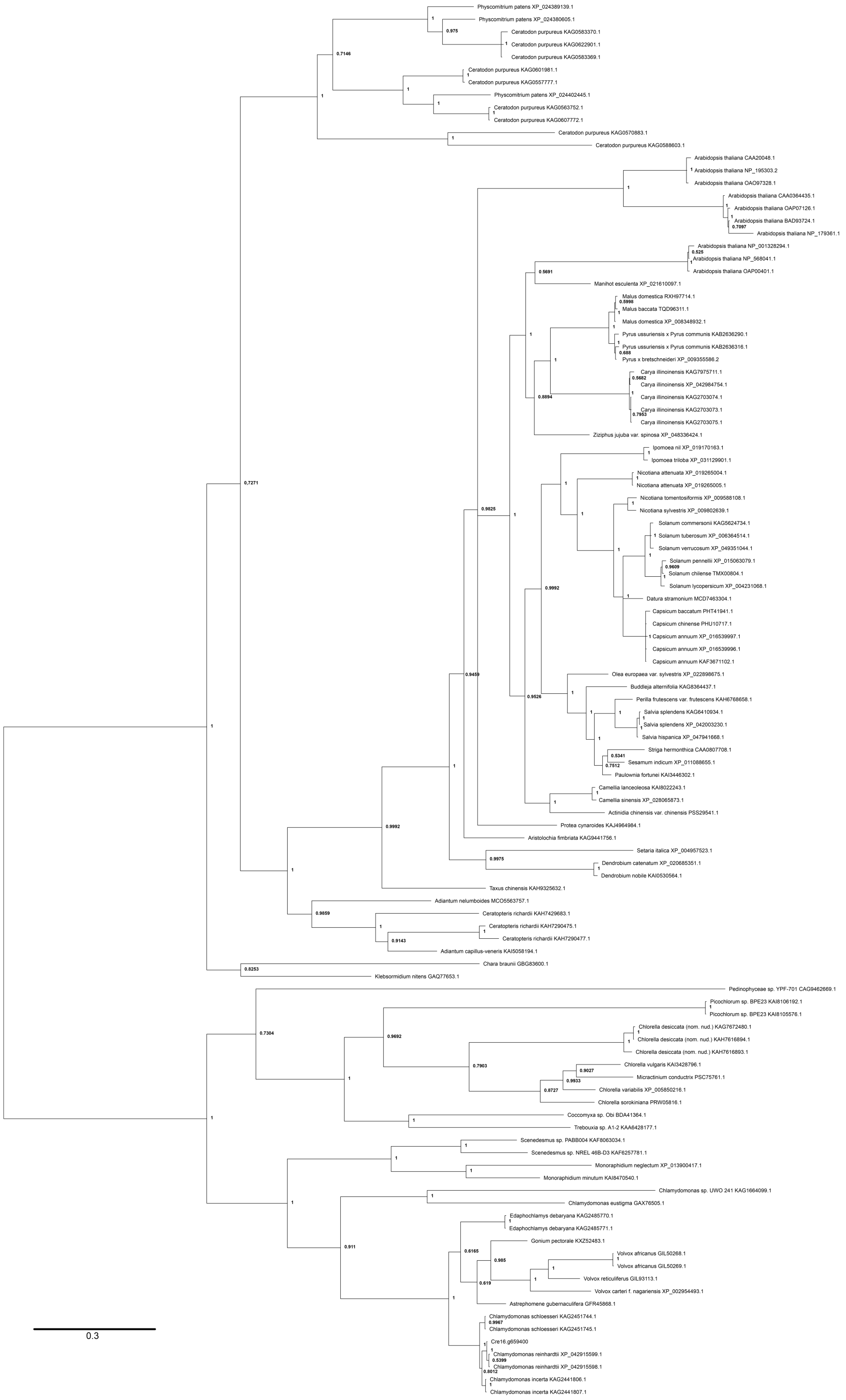

Supplementary Fig. 21. Phylogenetic tree of the PMSK-like family. Complete Unrooted phylogenetic tree of the PMSK-like family (dataset #1) identified as described in the "Identification of the PMSK-like family" from Chlorophyta representatives (Chlorophyceae, core Trebouxiophyceae, Chlorellales, Pedinophyceae), Charophyta, Briopsida, Pteridophyta, Gymnosperma and Angiosperma. The tree presented was inferred by Bayesian analysis as described in the "Methods" section. Bayesian Posterior Probability (BPP) values are reported at each node.

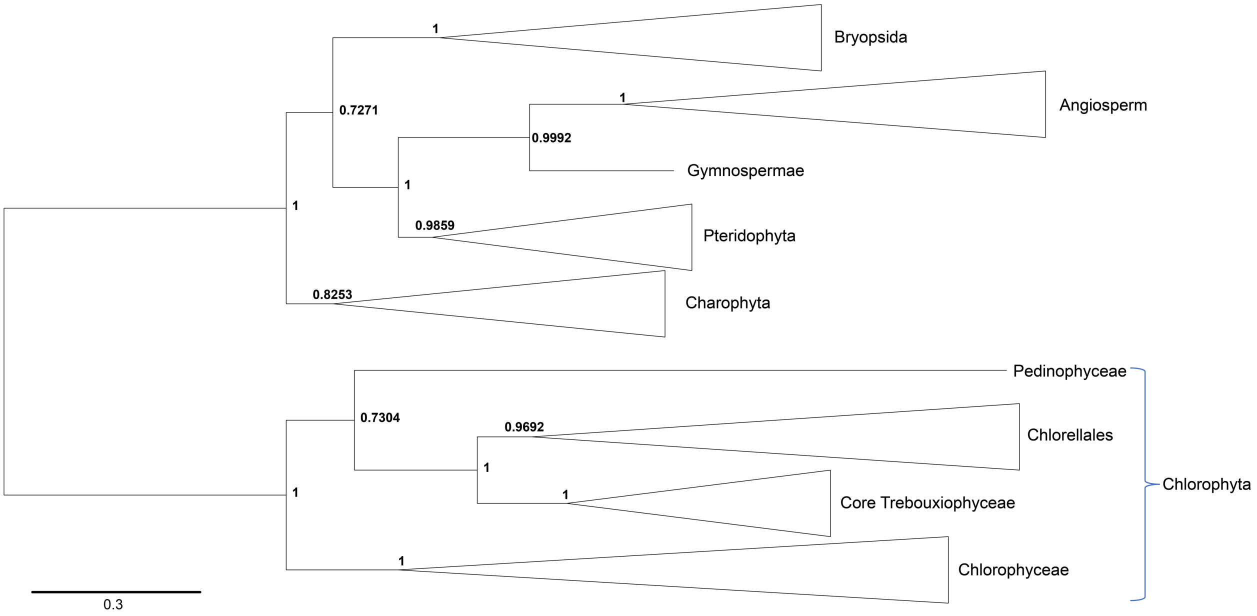

Supplementary Fig. 22. Evolutionary relationship between major green lineage clades inside the PMSK-like family. Simplified version of the **Supplementary Fig.21** phylogenetic tree of the PMSK-like family (dataset #1). The tree presented was inferred by Bayesian analysis as described in the "Methods" section. Bayesian Posterior Probability (BPP) values are reported at each node. This tree highlights the monophyletic origin of the PMSK family. The root represented on this tree is here to help the identification of major clades with the **Supplementary Fig.21** is not indicative of an ancestral state.

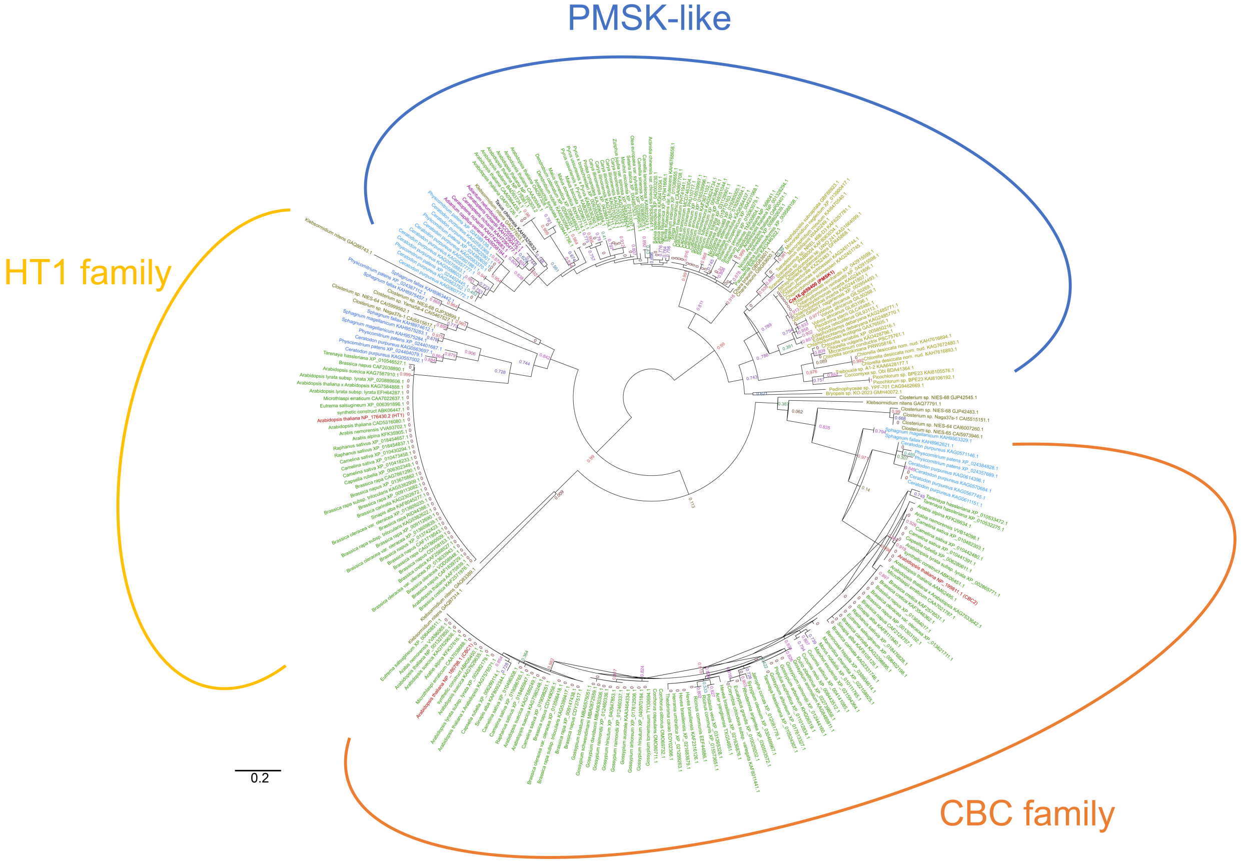

Supplementary Fig. 23. Evolutionary analysis by Maximum Likelihood method of the PMSK-like family with the HT1 and the CBC family

The evolutionary history was inferred from the dataset #2 by using the PhyML maximum likehood method from Chlorophyta representatives (Chlorophyceae, core Trebouxiophyceae, Chlorellales, Pedinophyceae), Charophyta, Briopsida, Pteridophyta, Gymnosperma and Angiosperma. The tree with the highest log likelihood (-4797.84912) is shown. The percentage of trees in which the associated taxa clustered together is shown next to the branches. Initial tree(s) for the heuristic search were obtained automatically by applying Neighbor-Join and BioNJ algorithms to a matrix of pairwise distances estimated using the WAG model, and then selecting the topology with superior log likelihood value. A discrete Gamma distribution was used to model evolutionary rate differences among sites (4 categories (+G, parameter = 1.283)). The rate variation model allowed for some sites to be evolutionarily invariable ([+I], 15.3% sites). The tree is drawn to scale, with branch lengths measured in the number of substitutions per site. This analysis involved 278 amino acid sequences. Evolutionary analyses were conducted in MEGA11.

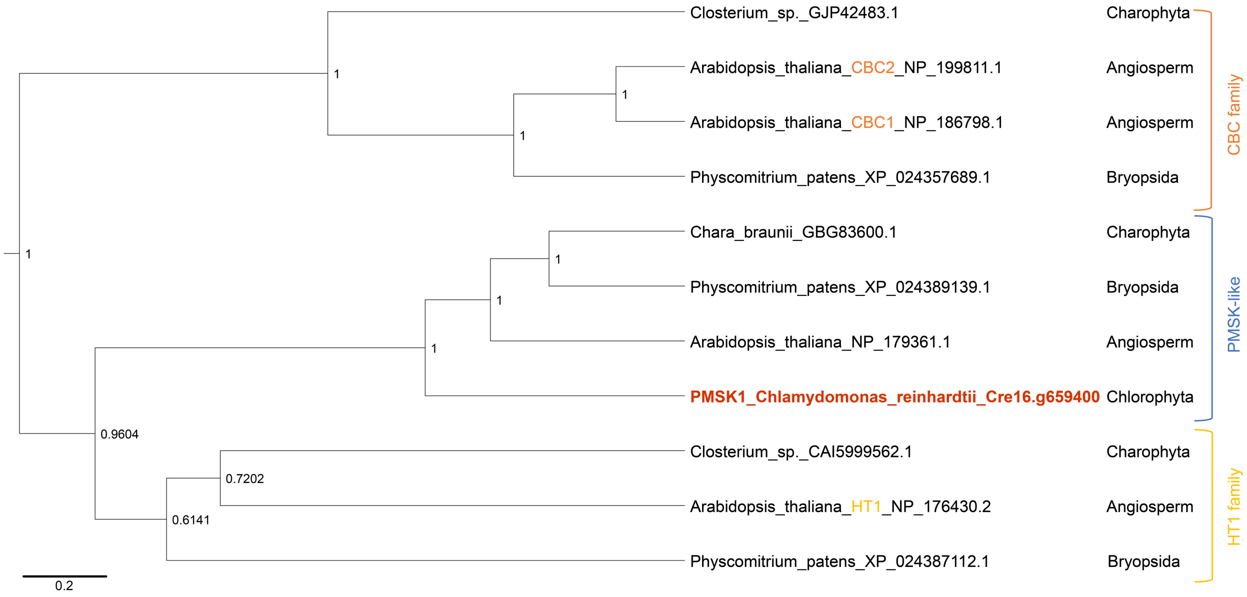

Supplementary Fig. 24. Phylogenetic clock tree of representatives of the PMSK-like, the HT1 and the CBC families. Rooted phylogenetic tree of representatives of the PMSK-like, the HT1 and the CBC families. The tree presented was inferred by Bayesian analysis with a clock-uniform method as described in the "Methods" section. Bayesian Posterior Probability (BPP) values are reported at each node. The root position was evaluated during the phylogenetic computation and is indicative of the evolutionary relationship between the three represented proteins families.

Supplementary References

1. Fang, L., Leliaert, F., Zhang, Z., Penny, D. & Zhong, B. Evolution of the Chlorophyta: Insights from chloroplast phylogenomic analyses. *J. Syst. Evol.* **55**, 322–332 (2017).

2. Sayers, E. W. *et al.* Database resources of the national center for biotechnology information. *Nucleic Acids Res.* **50**, D20–D26 (2021).

3. Altschul, S. F. *et al.* Gapped BLAST and PSI-BLAST: a new generation of protein database search programs. *Nucleic Acids Res.* **25**, 3389–3402 (1997).

4. Altschul, S. F. *et al.* Protein database searches using compositionally adjusted substitution matrices. *FEBS J.* **272**, 5101–5109 (2005).

5. Edgar, R. C. MUSCLE: multiple sequence alignment with high accuracy and high throughput. *Nucleic Acids Res.* **32**, 1792–1797 (2004).

6. Castresana, J. Selection of Conserved Blocks from Multiple Alignments for Their Use in Phylogenetic Analysis. *Mol. Biol. Evol.* **17**, 540–552 (2000).

7. Waterhouse, A. M., Procter, J. B., Martin, D. M. A., Clamp, M. & Barton, G. J. Jalview Version 2—a multiple sequence alignment editor and analysis workbench. *Bioinformatics* **25**, 1189–1191 (2009).

8. Ronquist, F. *et al.* MrBayes 3.2: Efficient Bayesian Phylogenetic Inference and Model Choice Across a Large Model Space. *Syst. Biol.* **61**, 539–542 (2012).

9. Whelan, S. & Goldman, N. A General Empirical Model of Protein Evolution Derived from Multiple Protein Families Using a Maximum-Likelihood Approach. *Mol. Biol. Evol.* **18**, 691–699 (2001).

10. Guindon, S. *et al.* New Algorithms and Methods to Estimate Maximum-Likelihood Phylogenies: Assessing the Performance of PhyML 3.0. *Syst. Biol.* **59**, 307–321 (2010).

11. Felsenstein, J. CONFIDENCE LIMITS ON PHYLOGENIES: AN APPROACH USING THE BOOTSTRAP. *Evolution* **39**, 783–791 (1985).

12. Anisimova, M. & Gascuel, O. Approximate Likelihood-Ratio Test for Branches: A Fast, Accurate, and Powerful Alternative. *Syst. Biol.* **55**, 539–552 (2006).
