# Supplementary material for: Phototropin connects blue light perception to starch metabolism in green algae
Source: Nat Commun. 2025 Mar 15;16:2545. doi: 10.1038/s41467-025-57809-3 (PMC11909140; doi:10.1038/s41467-025-57809-3)
Supplement: Supplementary file 3 — Description of Additional Supplementary Files [file 41467_2025_57809_MOESM3_ESM.pdf]

Description of Additional Supplementary Files

Supplementary Data 1. Oligo primers used in this study.

Supplementary Data 2. Proteomics data of WT and phot.

Supplementary Data 3. Phosphoproteomics data of WT and phot.

Supplementary Data 4. All Chlamydomonas reinhardtii strains used in this study
